# Langmuir-Blodgett Films of Arachidic and Stearic Acids as Sensitive Coatings for Chloroform HF SAW Sensors

**DOI:** 10.3390/s23010100

**Published:** 2022-12-22

**Authors:** Ilya Gorbachev, Andrey Smirnov, George Ivanov, Ivan Avramov, Elizaveta Datsuk, Tony Venelinov, Evgenija Bogdanova, Vladimir Anisimkin, Vladimir Kolesov, Iren Kuznetsova

**Affiliations:** 1 Kotelnikov Institute of Radio Engineering and Electronics of RAS, 125009 Moscow, Russia; 2 University Laboratory “Nanoscience and Nanotechnology”, University of Architecture, Civil Engineering and Geodesy, 1164 Sofia, Bulgaria; 3 G. Nadjakov Institute of Solid State Physics, Bulgarian Academy of Sciences, 1784 Sofia, Bulgaria

**Keywords:** Langmuir-Blodgett film, arachidic acid, stearic acid, chloroform sensor, Rayleigh SAW, two-port SAW resonators, quartz, SAW delay line

## Abstract

Properties of the Langmuir-Blodgett (LB) films of arachidic and stearic acids, versus the amount of the films’ monolayers were studied and applied for chloroform vapor detection with acoustoelectric high-frequency SAW sensors, based on an AT quartz two-port Rayleigh type SAW resonator (414 MHz) and ST-X quartz SAW delay line (157.5 MHz). Using both devices, it was confirmed that the film with 17 monolayers of stearic acid deposited on the surface of the SAW delay line at a surface pressure of 30 mN/m in the solid phase has the best sensitivity towards chloroform vapors, compared with the same films with other numbers of monolayers. For the SAW resonator sensing using slightly longer arachidic acid molecules, the optimum performance was reached with 17 LB film layers due to a sharper decrease in the Q-factor with mass loading. To understand the background of the result, Atomic Force Microscopy (AFM) in intermittent contact mode was used to study the morphology of the films, depending on the number of monolayers. The presence of the advanced morphology of the film surface with a maximal average roughness (9.3 nm) and surface area (29.7 µm^2^) was found only for 17-monolayer film. The effects of the chloroform vapors on the amplitude and the phase of the acoustic signal for both SAW devices at 20 °C were measured and compared with those for toluene and ethanol vapors; the largest responses were detected for chloroform vapor. For the film with an optimal number of monolayers, the largest amplitude response was measured for the resonator-based device. Conversely, the largest change in the acoustic phase produced by chloroform adsorption was measured for delay-line configuration. Finally, it was established that the gas responses for both devices coated with the LB films are completely restored 60 s after chamber cleaning with dry air.

## 1. Introduction

Environmental control of various chemical compounds is one of the main problems of modern ecology [1]. One such compound is chloroform, because it is frequently used in chemical industry and laboratories as a solvent [2,3] and penetrates easily into the human body with inhaled air. This gas acts on central nervous system and can accumulate in fat-rich tissues. With large amounts of chloroform entering the body, dystrophic changes in the internal organs, especially in liver, may appear. In this regard, the development of sensors controlling different concentrations of chloroform in the air is very important.

There are various physical approaches for developing chloroform sensors. They are based on field-effect transistors [4], resistivity elements [5], photoacoustic spectroscopy [6], optical fibers [7], etc. An alternative approach is the use of acoustic wave propagation in crystal substrates coated with appropriate sorbent films. This approach is based on the dependence of the acoustic wave properties (frequency, phase, amplitude) on the properties of the propagation medium, which change with gas-phase adsorption. Acoustic wave sensors are characterized by small size, low cost, reliability and high sensitivity to mass loading and change in the density, elasticity, electric conductivity and dielectric constants of sorbent films.

At present, few sensor configurations for detecting volatile organic compounds (VOCs), including chloroform, have already been developed. They are based on the bulk (BAW) and surface (SAW) acoustic wave resonators implemented on AT and ST quartz substrates, operating at 9 MHz to 435 MHz frequency range [8,9,10,11,12], the SAW delay lines (f = 38 MHz) implemented on lithium niobate crystal [13], the two-port SAW resonators (f = 434 MHz) [14,15,16,17], the film bulk acoustic resonators (FBAR) [18], the Love SAW devices [19], etc. The main problem for any sensor configuration and any type of sensor is a sorbent film whose physical properties should be changed remarkably and selectively in presence of chloroform vapor.

In the past, for registration of VOCs, in general, and chloroform, in particular, conducting polymer (CP) gate and magnesium silicide source heterojunction [4], copper oxide (CuO) nanoparticle and carbon nanotube (CNT) nanocomposite [5], modified cyclodextrines [8], metal phthalocyanines [9,13,20], electrospun ZnO nanostructured thin film [11], nanoporous-carbon coating [12], galix4arene modified gold nanorods (AuNRs) and silver nanocubes (AgNCs) [16], nanoporous gold sensitive layer [18], nanostructures of Sr doped WO3 [21] and calixarene [22] have been used as sorbent films. However, as before, selective detection of chloroform is still an unsolvable problem. 

In our previous work, Langmuir-Blodgett (LB) monolayer films fabricated from dipalmytoyl phosphatidyl ethanolamine head labeled with nitrbenzoxadiazole (DPPE-NBD) on a two-port SAW resonator (f = 411 MHz) [17,23] have demonstrated extremely high sensitivity to chloroform. Actually, the absorbed chloroform mass was bigger than the mass of the sensing monolayer mass, which is very rare [17]. However, it is known that phospholipids are difficult to deposit as a multilayer, which was no exception for the DPPE-NBD molecules [24]. Further, the price of around 500 EUR /1 mg of the DPPE-NBD molecule with high purity is prohibitive for mass production. The high sensitivity of the DPPE-NBD LB film monolayers can be explained by the well-developed 3D structure with a very high surface-to-volume ratio [25,26]. In a previous study, when the gas sensitivity of self-assembled molecules with different chemistry of the tails was compared among nine different types, the hydrocarbon CH_2_ group tales exhibited the second highest gas absorption properties [27]. As is known [28], stearic acid is in the same homologous series as arachidic acid, which differs from it in the number of carbon atoms in the hydrophobic part; additionally, chloroform is a good solvent for it. This result allows us to assume that Langmuir-Blodgett films based on it will be as sensitive to chloroform as films with arachidic acid. To prove this assumption, in this work, it was decided that we would use not only LB films with arachidic acid, but also LB films based on stearic acid as a sensor coating. 

In the present paper, we propose the use of Langmuir-Blodgett films of arachidic (AA) or stearic acids (SA) as sensing layers. We study the influence of the layer’s number on the morphology of the LB film of SA, and obtain evidence that the 17-layer films are optimum for sensing. This result was close to the optimum number of layers for LB film of AA reported earlier [17]. The concentration dependencies for chloroform are measured for two acoustic devices, namely, for a two-port Raleigh type SAW resonator (414 MHz) based on AT-quartz and for a SAW delay line (157.5 MHz) ST-X based on ST-quartz. The properties of the devices coated with the same films are compared with each other.

## 2. Materials and Methods

### 2.1. Formation of Sensitive Langmuir-Blodgett Films of Arachidic and Stearic Acids

The formation of a sensor coating based on a multilayer LB film of arachidic or stearic acid was carried out as follows. In the first step, AA or SA powder (Sigma-Aldrich, St. Louis, MO, USA, 99%) was dissolved in chloroform (Sigma Aldrich 99%). As a result, a solution of arachidic or stearic acid in chloroform with a concentration of 10^−3^ M/l was obtained. 

**Figure 1:**
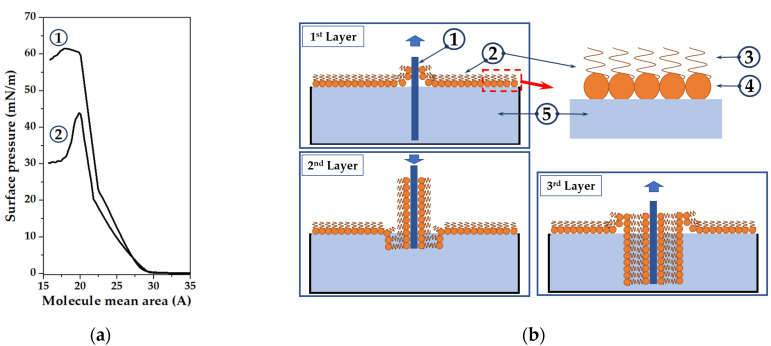
(**a**) Isotherm of compression of a monolayer of arachidic (1) and stearic (2) acids; (**b**) a schematic representation of the process of transferring a monolayer onto a solid substrate: (1) substrate, (2) AA or SA monolayer, (3) hydrophobic and (4) hydrophilic parts of AA or SA molecules, (5) water subphase.

The formation of the LB film was carried out using an LB Trough Nima KSV KN2001 setup (Biolin Scientific, Espoo, Finland) or Model 500 (Advanced Technologies Ltd., Sofia, Bulgary) at an aqueous subphase temperature of 22 °C and pH = 6.9. Deionized water with a resistivity of 18 MΩ × m was used as a subphase. Monolayers of arachidic or stearic acid were formed and transferred to solid substrates at the same compression rates of the monolayers. A 50 μL aliquot of a solution of AA or SA in chloroform was applied to the surface of the aqueous subphase. Evaporation of solvent molecules from the water surface took place within 20 min. Then, the monolayer was compressed by movable barriers at a constant area loss rate of 1 mm^2^/min. Figure 1 shows an isotherm of compression (a) of AA (1) and SA (2) monolayers and a schematic representation (b) of the process of monolayer transfer onto a solid substrate.

The transfer of the monolayer onto solid substrates was carried out according to the Langmuir-Blodgett method (vertical lift) (Figure 1b). The resonator surface was cleaned in air plasma at 18 W for 15 min before deposition. A SAW device was placed underwater to transfer the first layer of the AA or SA monolayer. This is because quartz and gold electrodes have a hydrophilic surface with a contact angle of less than 90° [29,30]. Next, a solution of AA or SA was applied to the surface of the water, and it was compressed by movable barriers until a surface pressure of 35 mN/m and 30 mN/m was achieved, respectively. The surface pressure of 35 mN/m and 30 mN/m for AA and SA, respectively, correspond to the middle point of the condensed state. The monolayer was transferred onto a solid substrate when the transfer pressure was reached. In this case, the substrate was lifted through the monolayer at a constant rate of 0.5 mm/min. Then, the substrate was dried for 5 min and again lowered through the monolayer under water. This process was repeated every 2.5 min. During the deposition of each subsequent layer, the time spent by the substrate above and below the water surface increased by 60 and 30 s, respectively. As a result, the monolayer transfer coefficient was 0.8. Thus, a sensor coating based on 17 monolayers of LB AA film was formed. As for LB SA film, 6 LB films were prepared consisting of 5, 9, 13, 17, 21, and 25 monolayers. The thicknesses of the ordered monolayer films of stearic and arachidic acids are 2.25 and 2.5 nm, respectively. With an increase in the number of layers in the film composition from 5 to 25, the film thickness will change in the range of 10 to 65 nm [31,32]. The surface morphology of the resulting layered LB SA films was studied further.

### 2.2. Study of Surface Morphology of Obtained Langmuir-Blodgett Films of Stearic Acid 

The surface morphology of the formed coatings based on LB SA films deposited onto the Si surface were studied by atomic force microscopy (AFM) using an NT-MDT Ntegra AFM probe microscope (NT-MDT, Zelenograd, Russia). *Si* substrate was chosen due to its availability, and it has a hydrophilic surface with a contact angle of less than 90°, like quartz. The scanning was carried out in a semi-contact mode with a frequency of 1 Hz. NSG01 probes (NT-MDT, Zelenograd, Russia) with a probe curvature radius of less than 10 nm were used for scanning. The obtained images with a resolution of 256 × 256 pixels were processed using the Gwyddion 2.61 software package [33,34] to calculate the roughness and surface area of the film. The film roughness was calculated by the formula:

(1) $$R_{a}=\frac{1}{N}\sum_{j=1}^{N}\left|r_{j}\right|$$

where *R_a_* is the arithmetic mean of the absolute values of the profile deviations within the base length (middle line of the profile), *N* is the number of points where the deviation is measured, and *r_j_* is the deviation of the absolute value from the midline of the profile at a point. 

### 2.3. Two-Port Rayleigh SAW Resonator

**Figure 2:**
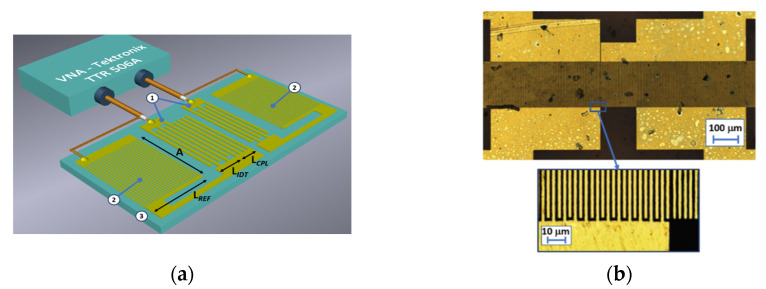
(**a**) Schematic view and (**b**) optical image of the central part of the device #1: 1: IDT, 2: reflector gratings, 3: piezoelectric substrate.

In this work, a two-port Rayleigh-type surface acoustic wave resonator (RSAW) implemented on AT, X-quartz (Eugler angles 0°, 125°, 0°) with gold electrodes and a central frequency of 414 MHz was used [17]. Figure 2 shows a schematic view (a) and an optical photo of the central part of the resonator (b) obtained by an optical confocal microscope Lext OLS5000 (Olympus Corp., Tokyo, Japan). This device #1 consists of two interdigital transducers (IDT) with the length of each IDT equal to *L_IDT_* = 500 μm and height of 100 nm, and two reflector gratings (RG) with the length of each RG *L_ref_* = of 2 mm, for generating and detection of an acoustic wave. The space between the transducers is completely occupied by the coupling grating (*L_cpl_* = 200 μm), which sets the number of longitudinal modes. Small space between the coupling grating and output IDT was chosen in such a way that one of the fundamental resonances was excited and detected with maximum efficiency and minimum losses, while the other modes were well suppressed [35,36,37,38]. The wavelength *λ*, aperture *A*, and substrate thickness were equal to 8 μm, 200 μm, and 0.5 mm, respectively. The electrodes were made of gold (100 nm) with a titanium sublayer.

The frequency dependencies of the insertion losses and phase of the acoustic signal were measured using a vector network analyzer TTR 506A (Tektronix, Beaverton, OR, USA). An OSLT compact calibration kit (4-in-1) 0–9 GHz N male BN 533884 (Spinner, Munich, Germany) was used for calibration.

**Figure 3:**
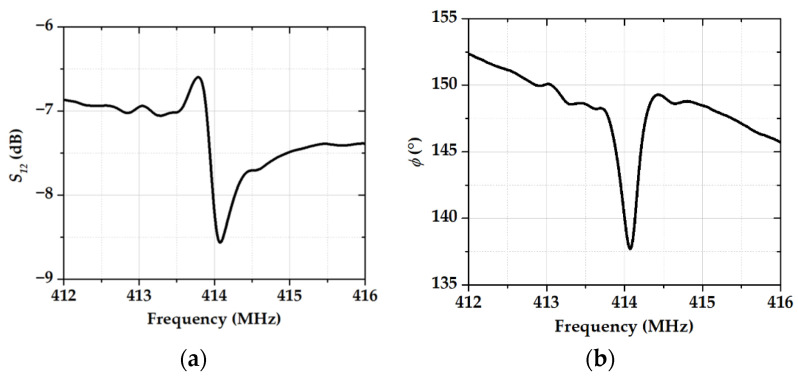
The frequency dependencies of (**a**) the *S_12_* parameter and (**b**) the phase for device #1 without sensitive film after surface plasma cleaning.

The frequency dependencies of the *S_12_* parameter and the phase of the acoustic signal for device #1 without sensitive film are shown in Figure 3.

**Figure 4:**
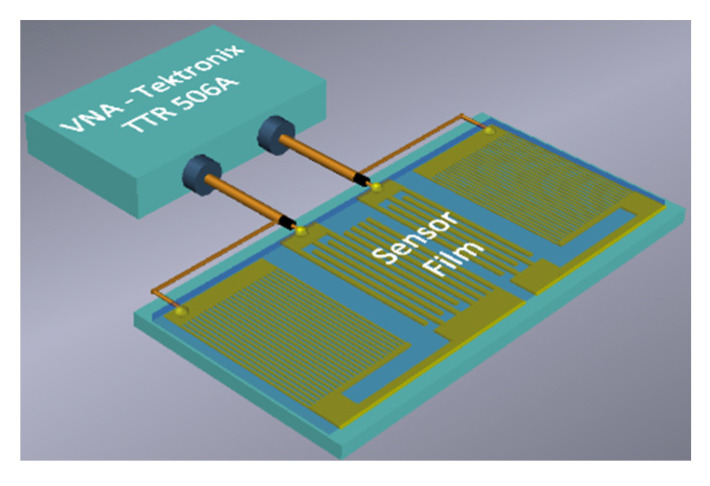
Schematic view of RSAW resonator (device #1) with the sensitive film over the whole propagation path.

**Figure 5:**
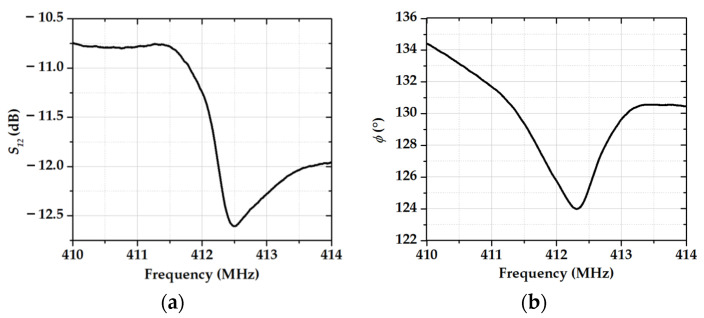
The frequency dependencies of (**a**) the *S_12_* parameter and (**b**) the phase for device #1, coated with LB film consisting of 17 monolayers of AA.

The sensitive LB film with AA was deposited over the whole surface of an RSAW resonator (*L_f_* ≈ *L_IDT_* + *L_cpl_*), as shown in Figure 4. The mass loading due to the film produces the change in the resonant frequency of device #1. Corresponding frequency dependencies of the *S_12_* parameter, and of the phase measured with LB film containing 17 monolayers of AA, are shown in Figure 5.

### 2.4. Rayleigh SAW Delay Line

The Rayleigh SAW delay line (device #2) operating at 157.5 MHz was based on polished ST-X quartz (Eugler angles 0°, 132.75°, 0°) with a thickness of 0.4 mm. The electrode structures were produced using maskless photolithography and magnetron sputtering. The magnetron was used in a direct current mode. 

**Figure 6:**
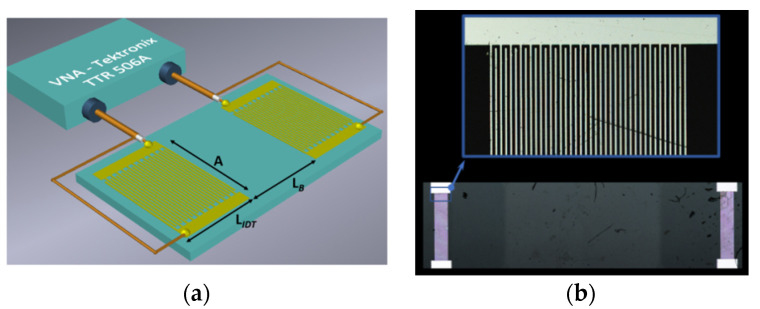
(**a**) Schematic view and (**b**) optical image of the Rayleigh SAW delay line (device #2).

The procedure was as follows. First, the layer of photoresist (S1813SP15) with a thickness of 0.02 mm was deposited on the piezoelectric plate surface, and then it was dried at the *T* = 96 °C. The electron photomask was produced by using a free program, Layout. The photomask obtained was projected on the plate surface with deposited photoresist using a set up Smart Print (Microlight 3D, La Tronche, France). The exposed part of the photoresist, which forms the electrode structure, was removed by the developer. Then, the plate was placed in a vacuum chamber of a magnetron sputtering unit VSE-PVD-DESK-PRO, and the plate surface was ionically cleaned in argon plasma during 10 s. After that, aluminum was deposited on the plate. During the deposition, the discharge power, deposition time, and pressure in the chamber were 200 W, 4 min, and 5.7 × 10^−3^ Torr, respectively. A film of chromium with a thickness of 5 nm was used as a sublayer. After deposition, the sample was cooled in the vacuum chamber in an inert argon atmosphere for 1 h. The rest of the photoresist was removed with acetone. As the result, an Al film with a thickness of 400 nm was obtained. A schematic view of device #2 connected to a vector analyzer, and its optical image obtained using a laser confocal microscope LEXT OLS5000 are presented in Figure 6. The wavelength *λ* is equal to 20 μm, the quantity of strips pair of each IDT *n* is equal to 20 (*L_IDT_* = *n* × *λ =* 400 μm), aperture *A* is equal to 2 mm, and the distances between transducers *L_B_* is equal to 8.2 mm. 

**Figure 7:**
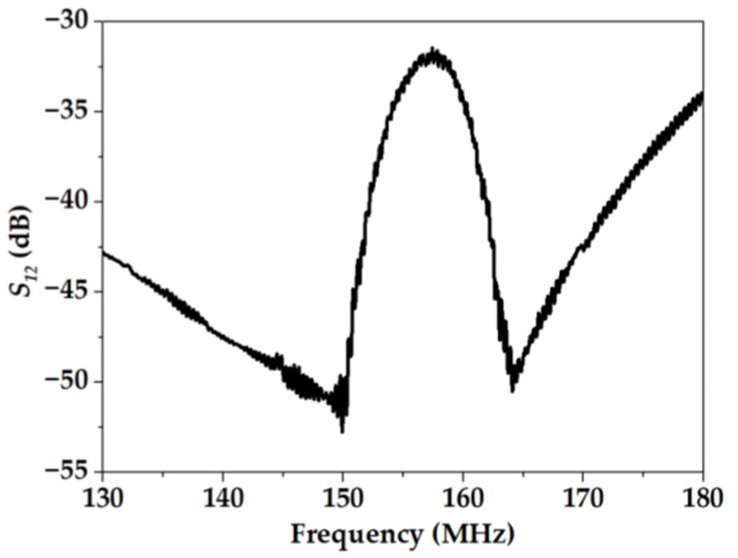
The frequency dependence of the *S_12_* parameter (insertion loss) for device #2 after plasma cleaning.

**Figure 8:**
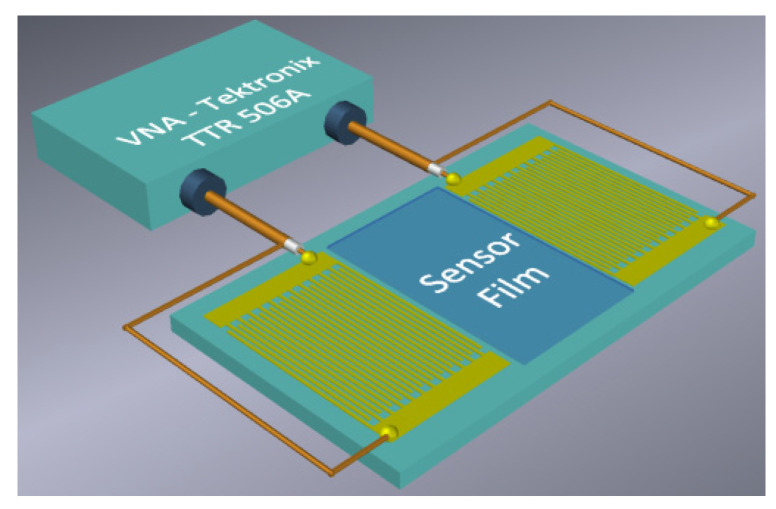
Schematic view of the Rayleigh SAW delay line with sorbent film between transducers.

The frequency dependence of the *S_12_* parameter was measured using the vector network analyzer (Tektronix TTR 506A). This dependence is presented in Figure 7. The sensitive film was placed between IDTs and has a length of about 8.2 mm, i.e., *L_f_* ≈ *L_B_* (Figure 8).

**Figure 9:**
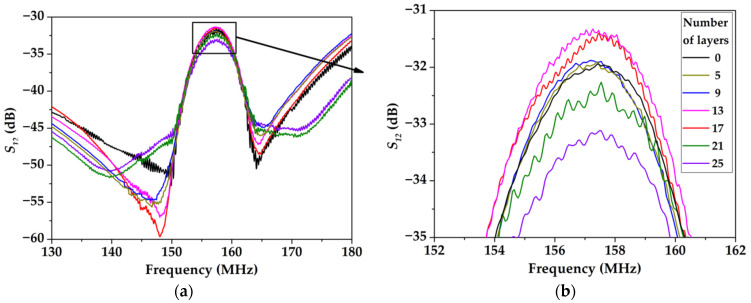
(**a**) The frequency dependencies of the *S_12_* parameter for device #2 loaded by LB film consisting of different number monolayers of SA; (**b**) enlarged fragment.

The frequency dependencies of the *S_12_* parameter for various numbers of monolayers of the LB SA film were measured using a vector network analyzer TTR 506A (Figure 9). It is seen that for the numbers of layers changing from 0 to 13, the insertion loss *S_12_* is decreased from −32 dB to −31.2 dB. On the contrary, for the number of layers changing from 13 to 25, the insertion loss S_12_ is increased from −31.2 dB to −33.1 dB (Figure 9b). The minimum insertion losses are measured for 0 to 25 monolayers.

This result may be explained as follows. Indeed, the whole insertion loss *S_12_* of the acoustic delay line is composed of (i) transduction losses in input and output IDTs, and (ii) propagation losses of the wave due to the viscosity of the film and the step between free and coated surfaces. Since LB film is a “slower” medium compared with quartz substrate, it traps the wave energy closer to the surface, increasing the wave localization near the surface for small film thickness. In this condition, the propagation losses of the wave and the insertion loss of the device are decreased with film thickness. On the other hand, when the thickness of the film is large enough, the scattering of the wave at the step and the wave attenuation in the film becomes too large, and the propagation losses of the wave and the insertion loss of the device are increased for thick films.

### 2.5. Set up and Methods for the Study of Gas-Sensitive Properties

**Figure 10:**
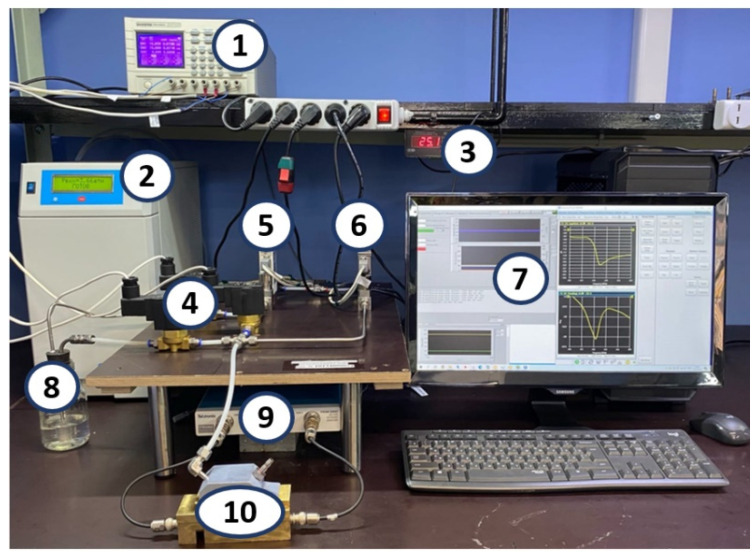
General view of the measuring setup. (1) 3-channel controlled power supply; (2) clean air generator; (3) chamber temperature and humidity meter; (4) electromechanical valves (5) Bronkhorst El-Flow Prestige flow meter; (6) Bronkhorst El-Flow flow meter; (7) personal computer with MOXA data acquisition board; (8) bubbler; (9) Tektronix TTR 506A vector network analyzer; (10) measuring chamber.

**Figure 11:**
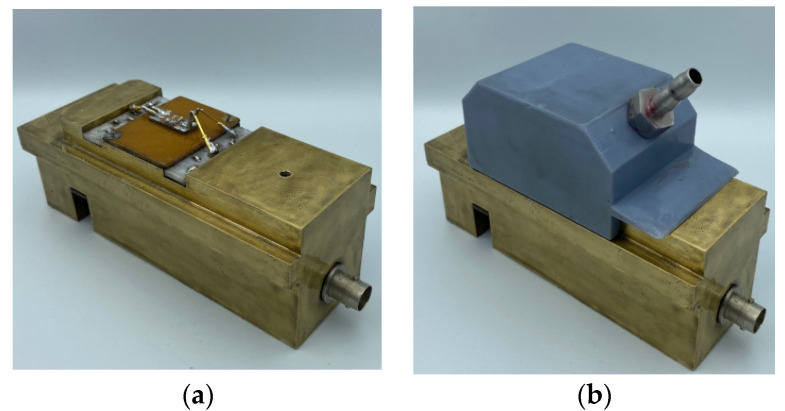
Photos of the sample holder: (**a**) without the gas chamber; (**b**) with the gas chamber.

The study of the gas-sensing properties of device #1 and device #2 at different concentrations of the target gases was carried out using an automated measuring setup. A general view of the used setup is shown in Figure 10. Photos of the sample holder without a gas chamber (a) and with a gas chamber (b) are shown in Figure 11. 

The stand was controlled and data were collected using the developed program. This program made it possible to set the measurement algorithm, i.e., valves were opened and closed at predetermined time intervals, and the supply and consumption of test gas samples were controlled. The clean air generator (2) was used to create a stream of clean and catalytically purified dry air, the moisture vapor content of which was no more than 10 ppm. 

**Figure 12:**
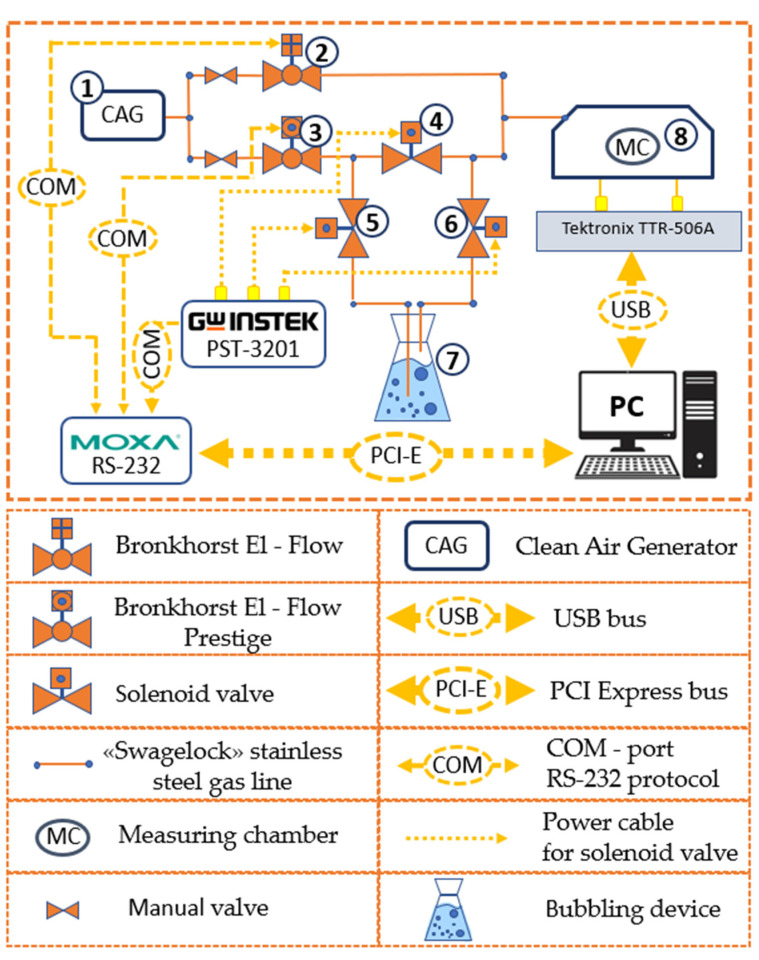
The scheme of an automated measuring setup.

A Bronkhorst EL-Flow Prestige flow meter (5) controlled the flow of dry air supplied to the test fluid bubbler (8). It was necessary for the creation of saturated steam. Flow control was carried out in the range from 0 to 100 sccm. A Bronkhorst EL-Flow (6) was used to purge the measurement chamber and dilute the test gas flow for the various concentrations obtained. Flow control was carried out in the range of 0–500 sccm. A three-channel controlled power supply (1) was used to open/close a solenoid valve (4). The flowmeters and the three-channel power supply were controlled using a MOXA CP-168U multiport board for the PCI bus via the RS-232 protocol. A glass container with a sealed, chemically resistant rubber stopper was used as the bubbler (8). A stainless steel stone diffuser with a pore diameter of 2 µm was used for aeration. This aerator was placed inside the bubbler (8). A thermohygrometer (3) was used to control humidity and temperature in the measuring chamber. Errors in measuring temperature and humidity were ±0.2 °C and ±2%, respectively. The compression fittings (Swagelok) and stainless steel and polytetrafluoroethylene pipes with a diameter of 6 mm were used to connect all elements of the gas system (Figure 12).

The air generator (1) generated a stream of clean, catalytically dried air. This air passed through the flow meters (2) and (3). The air passing through the flow meter (2) was used to pre-purge the measuring chamber (8) before the measurement. Valve (4) was closed, and valves (5) and (6) were opened for the creation of the required concentration of the test vapors. In this case, dry air through the flow meter (3) and valve (5) entered the bubbler (7). Intensive evaporation of the test substance occurred in the bubbler with a vapor concentration close to saturated vapor. Further, the resulting vapor of the test substance through the valve (6) entered the measuring chamber (8).

Thus, by changing the ratio of clean air and test vapor entering the chamber, different concentrations of test vapor were achieved. It is known that the concentration of vapor of the substance depends on atmospheric pressure, temperature, and the size of the bubbles in the bubbler. 

Due to the lack of calibrated sensors for chloroform, toluene, and ethanol vapors, an indirect method for determining the concentration of vapor of the substance in the measuring chamber was developed.

**Figure 13:**
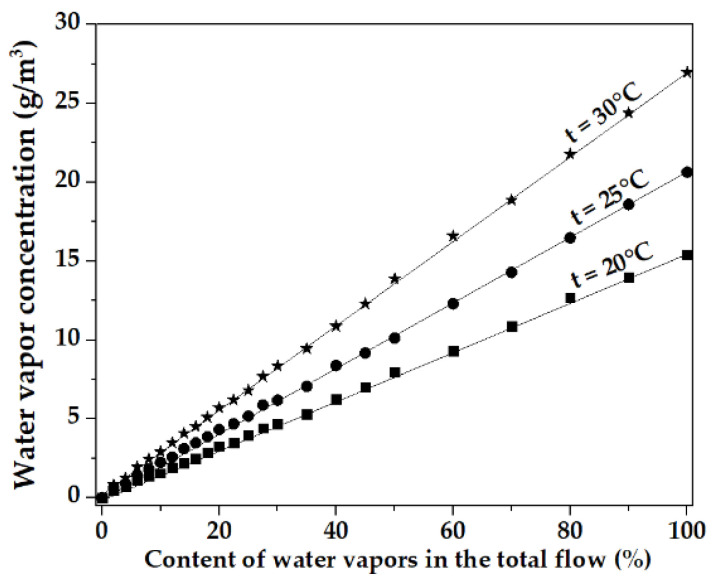
Dependencies of water vapor concentrations on their content in the measuring chamber at various ambient temperatures.

Distilled water was used for calibration. The dependences of water vapor concentration on their content were measured at ambient temperatures of 20 °C, 25 °C, and 30 °C (Figure 13). The temperature was controlled by a climatic system.

Figure 13 shows that the dependence of the concentrations of water vapor on their content in the measuring chamber is linear. It can be seen that when water vapor entered the measuring chamber from the bubbler at temperatures of 20 °C, 25 °C, and 30 °C, the water concentration inside the chamber was 15.39 g/m^3^, 20.63 g/m^3^, and 26.96 g/m^3^, respectively. 

This turned out to be equal to 89.4%, 89.6%, and 89.1% of the tabular values of the concentration of saturated water vapor at these temperatures, respectively. Thus, it was concluded that the maximum concentration of vapors of test substances obtained during bubbling is about 89% of the concentration of the saturated vapor. The Mendeleev-Claperon Formula (2) was used to determine the concentration of test substances’ vapors in the chamber, depending on the ratio of clean air and the test substances’ vapors:

(2) $$∁=n_{m}*m_{0}=\frac{P_{s}}{k*T}*\frac{M}{N_{A}}$$

where *C* is the concentration of the substance in g/m^3^, *n_m_* is the number of molecules per unit volume in m^−3^, *m*_0_ is the mass of one molecule in g, *P_S_* is the saturation vapor pressure in Pa, *k* is the Boltzmann constant, *T* is the absolute temperature in K, *M* is molar mass and *N_A_* is Avogadro’s number.

The concentration of the saturated vapors of the test substances, calculated by (2), was equal to 1017.99, 111.41, and 108.15 g/m^3^ for chloroform, ethanol, and toluene, respectively. The measured concentration values in the measuring chamber were obtained by multiplying the maximum vapor concentrations of the test substances by the factor 0.89 obtained above. The data presented in Table 1 were obtained assuming a linear dependence of the test substance vapor concentration on the ratio of clean air and test substance vapor in the measuring chamber.

### 2.6. Method for Calculation of Acoustoelectronic Devices Responses

Responses of the acoustoelectronic devices #1 and #2 were determined to be similar to those [39]. 

The amplitude response was defined as:

(3) $$\alpha =\frac{\Delta S_{12}}{L},\;[\mathrm{dB}/\mathrm{mm}],$$

where Δ*S*_12_ is the change in the signal amplitude at the center frequency produced by gas adsorption, *L* = *L_IDT_* + *L_CPL_* for device #1 and *L* = *L_f_* for device #2.

(4) $$R=\frac{\Delta \varphi }{\varphi_{0}}\times 10^{6},\;[\mathrm{ppm}],$$

where Δ*φ* is the change in the acoustic phase due to gas adsorption measured at the center frequency for each device, and *φ*_0_ = 360° *L*/*λ* is the total SAW phase acquiring the wave when propagating between input and output transducers. For device #1, where the sorbent film is over the whole propagation path, Δ*φ* is just the value measured with the network analyzer, and *φ_0_* is calculated for *L* = *L_IDT_* + *L_CPL_*. For device #2, where the sorbent film is deposited only between transducers, the measured value Δ*φ* is increased by a factor (*L_IDT_* + *L_f_*) × L_f_, and *φ_0_* is calculated using *L* = *L_IDT_* + *L_B_* ≈ *L_IDT_* + *L_f_*.

## 3. Results and Discussion

### 3.1. The Surface Morphology of Multilayer Coatings Based on LB Films of Stearic Acid

The roughness values *R_a_* for LB films with SA were calculated as the arithmetic means of the R_a_ parameter using Formula (1) and seven surface images with a resolution of 5 × 5 µm. The obtained results for the LB SA films with different numbers of monolayers are presented in Table 2.

**Figure 14:**
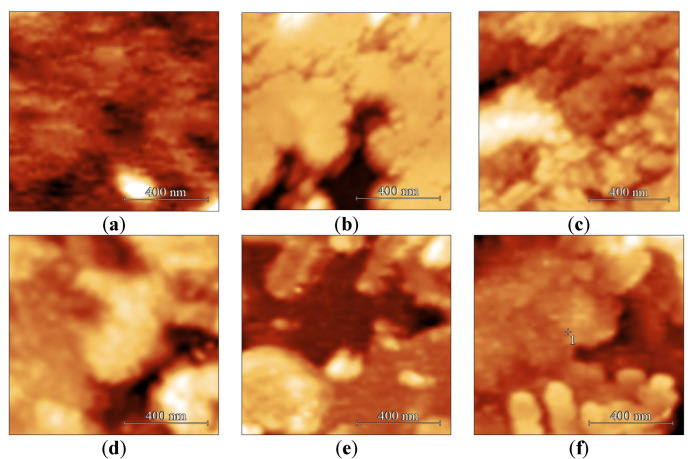
AFM images of the surface morphology of LB films containing (**a**) 5; (**b**) 9; (**c**) 13; (**d**) 17, (**e**) 21; and (**f**) 25 monolayers of stearic acid made at a surface pressure of 30 mN/m and placed on *Si* substrate.

AFM images of the surface of LB SA films with different numbers of monolayers with a size of 1 × 1 µm placed on *Si* substrate are shown in Figure 14.

From Table 2 and Figure 14a, it is seen that the resulting 5-layer stearic acid film has a developed morphology. The islands with a diameter of about 0.4–0.8 μm and a height of 2–6 nm can be distinguished in the film structure.

The height of individual islands is comparable to 2–3 lengths of a stearic acid molecule oriented perpendicular to the surface. An increase in the number of layers in the film to 9 increases the size of SA islands, and in some areas they form a continuous film with defects. In this case, the average film roughness slightly increases from 5.1 to 5.3 nm, and the film surface area increases from 27.8 μm^2^ to 28.1 μm^2^. A further increase in the number of layers in the film to 13 leads to the formation of a continuous film with defects. In this case, the film roughness and its surface area increase to 6.7 nm and 28.4 µm^2^, respectively. Such behavior of these parameters is associated with an increase in the degree of inhomogeneity of the film surface. Roughness and surface area are 9.3 nm and 29.7 µm^2^, respectively, for a 17-layer film. A further increase in the number of layers in the film leads to a decrease in both its roughness and surface area (Table 2). Such behavior can be explained as follows.

**Figure 15:**
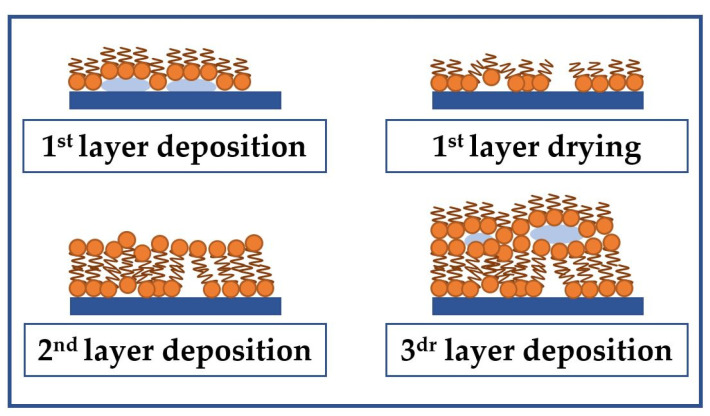
Schematic presentation of the formation of an LB film of stearic acid with a developed morphology on the surface of a hydrophilic substrate.

As is known [40], the method of transfer of LB films and their morphology strongly depends on the wetting angle of the used substrate. For quartz, this value is 65°. Silicon was used as a substrate to study the effect of the number of monolayers of the stearic acid LB film on its morphology. For this material, the contact angle is also 65°, and it is more accessible for a series of experiments. Due to the hydrophilicity of the substrate used, the formation of the first monolayer of stearic acid is performed by carrying the substrate from the subphase into the air. Thus, the hydrophilic parts of the stearic acid molecules are oriented toward the plate. Further monolayers are formed by carrying the substrate out of the air into the subphase. The formation of film inhomogeneities can be associated with the escape of water molecules from the space between the film and substrate during drying. In addition, when the layers interact with each other, the appearance of crystallization centers and the formation of nanocrystallites are possible. The appearance of such nanocrystallites can also be one of the reasons for the increase in surface roughness. Subsequent monolayers cover the formed inhomogeneities in the film, which leads to an increase in the degree of its roughness and the appearance of a developed morphology. The process of formation of such a structure is schematically shown in Figure 15. This process is observed for films containing up to 17 monolayers. With a further increase in the number of monolayers, the effect of adsorbed water molecules and the resulting nanocrystallites on the morphology of the resulting film decreases. As a result, the roughness and surface area of the film also decreases.

### 3.2. The Gas-Sensitive Properties of the Sensors Based on RSAW Resonator with LB Film of AA and SAW Delay Line with LB Film of SA

**Figure 16:**
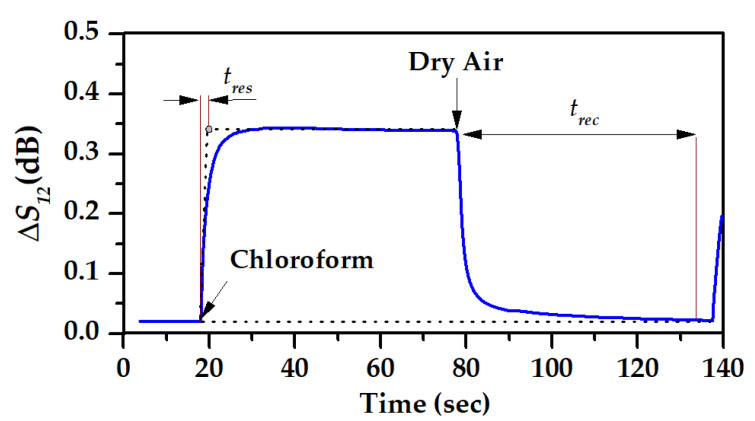
Time dependence of Δ*S_12_* during the admission of saturated chloroform vapors and further blowing with dry air.

The gas sample was fed into the measuring chamber according to Table 1. The exposure time at each point sufficient to reach saturation was 5 min. After the end of each stage of the experiment, the measuring chamber was purged with a stream of dry air. It was found that the time to return the frequency and responses to the initial values is about 60 s after the start of blowing with dry air. Response of the resonator to saturated chloroform vapors was within seconds and was measured every 50 ms [23]. A typical graph of the time dependence of Δ*S*_12_ during the admission of saturated chloroform vapors and further blowing with dry air is shown in Figure 16. The response time (*t_res_*) and recovery time (*t_rec_*) were about 1.2 s and no more than 60 s, respectively. 

**Figure 17:**
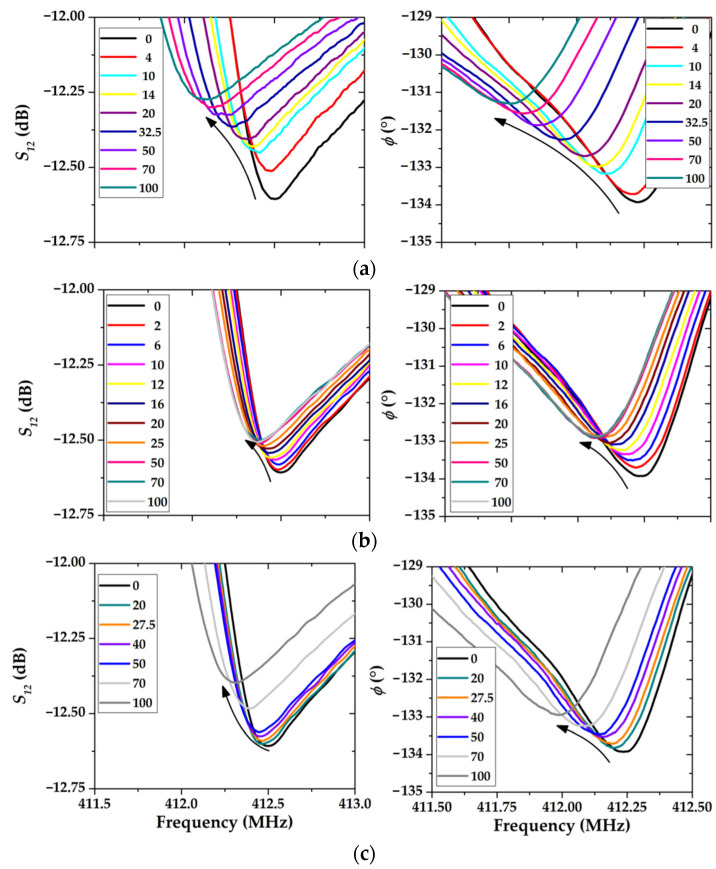
Frequency dependencies of the *S_12_* parameter and the phase *φ* of device #1 with LB consisting of 17 monolayers of AA-sensing layer film at different gas concentrations from 0 to 100%. (**a**) chloroform; (**b**) toluene; (**c**) ethanol.

The results of experiments to study the effect of chloroform, toluene, and ethanol vapors on the frequency and phase responses of the HF RSAW resonator (device #1), with sensitive LB film consisting of 17 monolayers of AA, are shown in Figure 17.

**Figure 18:**
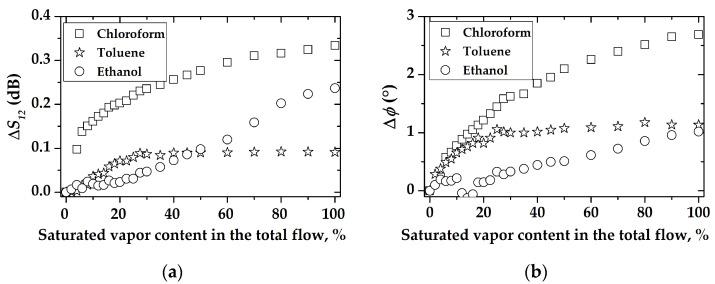
The changes in the positions of the extrema from Figure 16 for (**a**) *S_12_* parameter and (**b**) phase *φ*, versus different gases concentrations for device #1.

**Figure 19:**
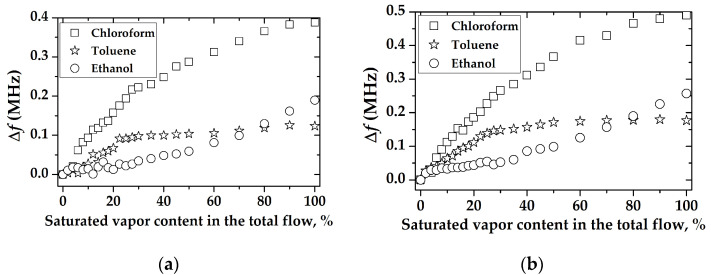
The changes in the frequency of the extrema from Figure 16 for (**a**) the *S_12_* parameter and (**b**) phase *φ*, versus different gas concentrations for device #1.

The auxiliary graphs that describe changes in the position of extrema of (a) *S*_12_ and (b) *φ* with a change in the gas concentration are presented in Figure 18. It can be seen that the greatest changes in the position of the resonance are measured only for chloroform vapors. As the chloroform concentration changed from 18.24 to 912.12 g/m^3^, the value of the *S_12_* parameter at the resonance changed about 0.33 dB. At the same time, the position of the resonant frequency shifted down to about 387 kHz (Figure 19a). Similar behavior is observed for the frequency phase characteristics; maximal phase shift is about 2.68° (Figure 18b) and relevant resonant frequency change is 488 kHz (Figure 19b).

The behavior of the concentration dependence of toluene vapor differs significantly from that of chloroform (Figure 17b). For example, the maximum response for toluene in Figure 17a is 0.09 dB, which is 3.5 times less than that for chloroform. Additionally, the maximum change in the position of the resonance is observed in the range of 0–30% of the content of saturated toluene vapor in the total gas flow. A further increase in the toluene concentration has practically no effect on the position of the peak. This allows us to conclude that the operating range of the sensor is limited by the maximum concentration of toluene vapor 25–30 g/m^3^. 

The maximum peak shift in the frequency response when exposed to the maximum concentration of the ethanol sample is 0.23 dB and 189 kHz (Figure 18a and Figure 19a), on the phase frequency characteristics 1° and 257 kHz, respectively (Figure 18b and Figure 19b). It can be seen that in the ethanol concentration range of 0–30% of saturated vapor, the position of the peak practically does not change, and the operating range of such a sensor is 30–100%.

Analysis of Figure 17, Figure 18 and Figure 19 has shown that for device #1, the sensitive coating based on LB film with 17 monolayers of AA is more sensitive to chloroform vapor than to toluene or ethanol vapors. This may be due to the high affinity of chloroform and arachidic acid molecules compared with pairs of other substances. Hansen’s solubility parameters can be used to determine the degree of affinity of a substance’s molecules with a solvent. These parameters make it possible to estimate the contribution of dispersion (*δ_d_*) and dipole (*δ_p_*) interactions, as well as hydrogen bonds (*δ_H_*) to solubility. The corresponding Hansen parameters for vapors of chloroform, toluene, ethanol, as well as arachidic and stearic acids are given in Table 3 [41,42].

The relative energy difference (*RED*) parameter is used to quantify the dissolving power of vapor with respect to the dissolved material (acid):

(5) $$RED=\frac{R_{a}^{vapor-acid}}{R_{0}}.$$

Here, *R_0_* is the radius of interaction between molecules of arachidic and stearic acids, equal to 3.99 and 4.7, respectively [43].

(6) $$R_{a}^{vapor-acid}=\sqrt{4{\left(\delta_{di}-\delta_{dj}\right)}^{2}+{\left(\delta_{pi}-\delta_{pj}\right)}^{2}+{\left(\delta_{Hi}-\delta_{Hj}\right)}^{2}}.$$

Here, *δ_pi_*, *δ_di_*, *δ_Hi_*, *δ_pj_*, *δ_dj_* and *δ_Hj_* are the Hansen solubility parameters for vapor and acid. Indexes i and j correspond to vapor and acid, respectively. The calculated values of $R_{a}^{vapor-acid}$ are presented in Table 4.

Table 4 shows that for a pair of chloroform-SA or chloroform-AA, *RED* < 1. This means that chloroform is the most suitable solvent for arachidic or stearic acid. Moreover, these acids are insoluble in ethanol and only partially soluble in toluene.

The comparison has shown that stearic acid has practically the same Hansen solubility parameters as arachidic acid. So, stearic acid could be used to study the influence of the monolayer amount on chloroform sensitivity.

After finishing experiments with device #1 based on an RSAW resonator with LB film consisting of 17 monolayers of AA, the study of gas sensitivity of device #2, based on RSAW delay line with LB film consisting of various numbers (5–25) of SA monolayers, was performed.

**Figure 20:**
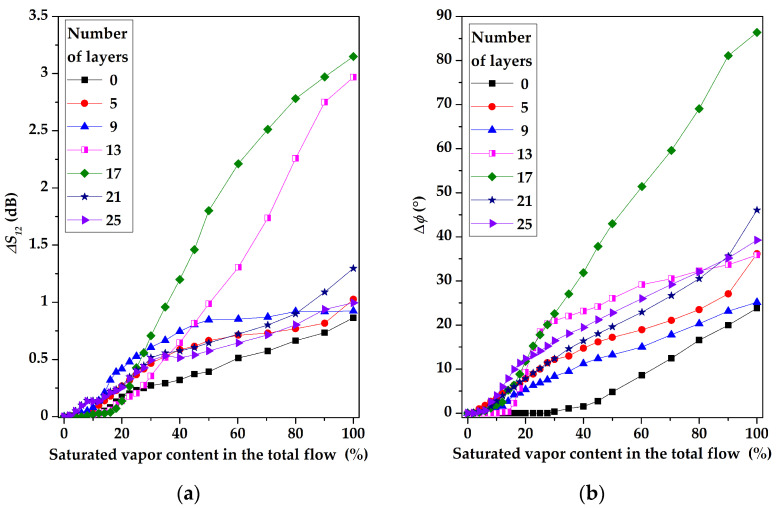
The change in (**a**) the S_12_ parameter and (**b**) phase for device #2 coated with LB SA-sensitive film versus chloroform concentration was measured for different numbers of SA monolayers in LB SA-sensitive film.

The dependences of the changes in *S_12_* parameter (a) and phase (b) versus the content of chloroform vapor in the total gas flow (chloroform concentration) are shown in Figure 20. It is seen that the largest change in *S_12_* and the phase was measured only for the film consisting of 17 SA monolayers. This result confirms previous conclusions and results reported earlier in the paper [17].

**Figure 21:**
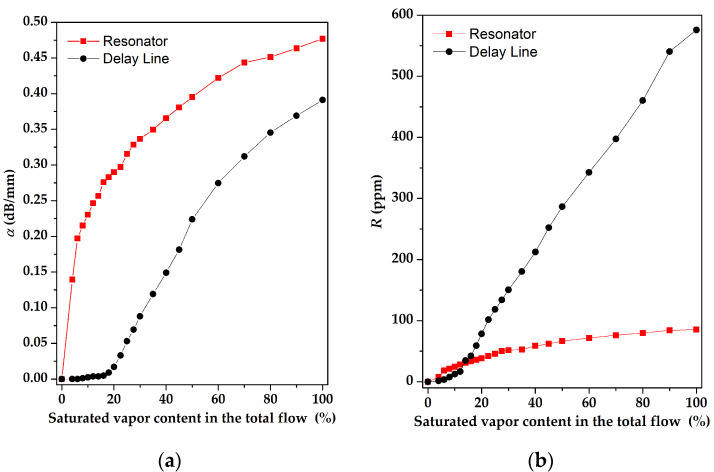
(**a**) The amplitude and (**b**) the phase responses of devices #1 with LB AA and #2 with LB SA-sensitive films consisting of 17 monolayers versus chloroform concentration.

The comparison of the sensitivity of the sensors based on a two-port RSAW resonator (f = 414 MHz) and RSAW delay line (f = 157.5 MHz) with LB AA and LB SA of 17 monolayers films, respectively, to various concentrations of chloroform, is presented in Figure 21.

Figure 21 shows that the amplitude of gas response for the SAW resonator is larger than that for the SAW delay line. On the other hand, the phase response of the SAW delay line configuration at high concentrations is dominant, compared with the SAW resonator. We attribute these properties to different topologies of the SAW devices. The resonator devices are extremely sensitive towards SAW attenuation increased with mass loading; the delay lines are more sensitive towards phase variations as they have a much larger total SAW phase *φ_0_* acquiring the wave between input and output transducers. For example, the values of *φ_0_* for devices #1 and #2 used in our paper are 31,500 grads and 154,800 grads, respectively. Moreover, an additional contribution to the phase response may be due to the change in the film conductivity (if any) producing an additional impact on the phase variations.

## 4. Conclusions

Based on two different SAW devices (two-port resonator and common delay line) the applicability of the Langmuir-Blodgett films of arachidic and stearic acids as sensitive coatings for chloroform detection is demonstrated. The optimal number of the mono-layers for the films is found as 17, when they possess maximal average roughness (9.3 nm) and surface area (29.7 µm^2^). For the films with the optimal number of mono-layers, both amplitude and phase responses of the SAW sensors towards chloroform are a few times larger than those for toluene and ethanol. All gas responses are reversible for about 60 s after film cleaning with dry air. It is found also that when the amplitude response is preferable for application, the film-coated SAW resonators are more attractive. On the other hand, when the phase responses are convenient, the delay line configuration with the sorbent Langmuir-Blodgett film located in the gap between input and output transducers is more suitable.

## Figures and Tables

**Table 1:** Vapor concentration of the test substances at 20 °C.

Content Probe in Total Flow, %	Chloroform (*CHCl*_3_), g/m^3^	Ethanol (*C*_2_*H*_5_*OH*), g/m^3^	Toluene (*CH*), g/m^3^
2	18.24	1.99	1.93
4	36.48	3.99	3.87
6	54.72	5.98	5.81
8	72.97	7.98	7.75
10	91.21	9.98	9.69
12	109.45	11.97	11.62
14	127.69	13.97	13.56
16	145.94	15.96	15.51
18	164.18	17.96	17.44
20	182.42	19.96	19.38
22.5	205.22	22.45	21.81
25	228.03	24.95	24.22
27.5	250.56	27.54	26.75
30	274.67	30.27	29.25
35	319.35	35.08	34.16
40	364.85	39.92	38.76
45	410.54	45.24	43.89
50	456.06	49.91	48.45
60	545.58	60.12	58.24
70	639.89	70.17	68.09
80	731.63	80.14	77.87
90	822.74	90.16	87.45
100	912.12	99.81	96.91

**Table 2:** Roughness parameters and surface areas of the LB SA films with different numbers of monolayers.

Number of Layers, pcs.	*R_a_*, nm	Surface Area, μm^2^
5	5.1	27.8
9	5.3	28.1
13	6.7	28.4
17	9.3	29.7
21	7.6	27.5
25	7.3	26.2

**Table 3:** Hansen solubility parameters for vapors of chloroform, toluene, ethanol, as well as arachidic and stearic acids [41,42].

Material	δ_d_, MPa^1/2^	δ_p_, MPa^1/2^	δ_H_, MPa^1/2^
Chloroform	17.8	3.1	5.7
Ethanol	15.8	8.8	19.4
Toluene	18	1.4	2
Arachidic acid	16.3	2.9	5
Stearic acid	16.3	3.3	5.5

**Table 4:** The calculated values of $R_{a}^{vapor-acid}$.

		$R_{a}^{vapor-acid},$ MPa^1/2^		*RED*	
	Acid	SA	AA	SA	AA
Vapor					
Chloroform		3	3.1	0.6	0.8
Ethanol		14.9	15.5	3.2	3.9
Toluene		5.2	4.8	1.1	1.2

## Data Availability

No new data were created or analyzed in this study. Data sharing is not applicable to this article.

## References

[1] Gavrilescu M., Demnerová K., Aamand J., Agathos S., Fava F. (2015). Emerging pollutants in the environment: Present and future challenges in biomonitoring, ecological risks, and bioremediation. New Biotechnol..

[2] Jordan A., Stoy P., Sneddon H.F. (2020). Chlorinated solvents: Their advantages, disadvantages, and alternatives in organic and medicinal chemistry. Chem. Rev..

[3] Joshi D.R., Adhikari N. (2019). An overview on common organic solvents and their toxicity. J. Pharm. Res. Int..

[4] Dassi M., Madan J., Pandey R., Sharma R. (2022). Chemical modulation of conducting polymer gate electrode work function based double gate Mg_2_Si TFET for gas sensing applications. J. Mater. Sci. Mater. Electron..

[5] Onthath H., Maurya M.R., Bykkam S., Kasak P., Sadasivuni K.K. (2021). Development and Fabrication of Carbon Nanotube (CNT)/CuO Nanocomposite for Volatile Organic Compounds (VOCs) Gas Sensor Application. Macromol. Symp..

[6] Zhang T., Xing Y., Wang G., He S. (2021). High sensitivity continuous monitoring of chloroform gas by using wavelength modulation photoacoustic spectroscopy in the near-infrared range. Appl. Sci..

[7] Tapanes E.E., Goode J.R., Rossiter P.L., Hill A.J. (1995). Evanescent wave spectroscopy as an interface probe. Materials Science Forum.

[8] Dickert F.L., Bruckdorfer T., Feigl H., Haunschild A., Kuschow V., Obermeier E., Bulst W.E., Knauer U., Mages G. (1993). Supramolecular detection vapours with QMB and SAW devices. Sens. Actuators B Chem..

[9] Fernandes D.L.A., Gomes M.T.S.R. (2008). Development of an electronic nose to identify and quantify volatile hazardous compounds. Talanta.

[10] Singh P., Yadava R.D.S. (2011). Effect of film thickness and viscoelasticity on separability of vapour classes by wavelet and principal component analyses of polymer-coated surface acoustic wave sensor transients. Meas. Sci. Technol..

[11] Tasaltin C., Ebeoglu M.A., Ozturk Z.Z. (2012). Acoustoelectric effect on the responses of SAW sensors coated with electrospun ZnO nanostructured thin film. Sensors.

[12] Siegal M.P., Mowry C.D., Pfeifer K.B., Gallis D.F.S. (2015). Detecting trihalomethanes using nanoporous-carbon coated surface-acoustic-wave sensors. J. Electrochem. Soc..

[13] Urbańczyk M., Jakubik W., Kochowski S. (1994). Investigation of sensor properties of copper phthalocyanine with the use of surface acoustic waves. Sens. Actuators B Chem..

[14] Avramov I.D., Stahl U. On the Mass Sensitivity of Rayleigh Surface Acoustic Wave (RSAW) Resonators. Proceedings of the 2017 40th International Spring Seminar on Electronics Technology (ISSE).

[15] Stahl U., Voigt A., Dirschka M., Barié N., Richter C., Waldbaur A., Gruhl F.J., Rapp B.E., Rapp M., Länge K. (2017). Long-term stability of polymer-coated surface transverse wave sensors for the detection of organic solvent vapors. Sensors.

[16] Kus F., Altinkok C., Zayim E., Erdemir S., Tasaltin C., Gurol I. (2021). Surface acoustic wave (SAW) sensor for volatile organic compounds (VOCs) detection with calix[4]arene functionalized Gold nanorods (AuNRs) and silver nanocubes (AgNCs). Sens. Actuators B Chem..

[17] Avramov I.D., Ivanov G.R. (2022). Layer by layer optimization of Langmuir–Blodgett films for surface acoustic wave (SAW) based sensors for volatile organic compounds (VOC) detection. Coatings.

[18] Guo H., Gao Y., Liu T. (2018). A theoretical study of the VOC sensor based on polymer-coated diaphragm embedded with FBAR. Measurement.

[19] Viespe C., Dinca V., Popescu-Pelin G., Miu D. (2019). Love wave surface acoustic wave sensor with laser-deposited nanoporous gold sensitive layer. Sensors.

[20] Kılınc N., Atilla D., Gurek A.G., Ozturka Z.Z., Ahsen V. (2009). Volatile organic compounds sensing properties of tetrakis(alkylthio)-substituted lutetium(III) bis phthalocyanines thin films. Talanta.

[21] Adimule V., Bhowmik D., Gowda A.H.J. (2021). Morphology, Characterization, and Gas Sensor Properties of Sr Doped WO3 Thin Film Nanostructures. Macromolecular Symposia.

[22] Schierbaum K.-D., Gerlach A., Gopel W., Muller W.M., Vogtle F., Dominik A., Roth H.J. (1994). Surface and bulk interactions of organic molecules with calixarene layers. Fresenius’ J. Anal. Chem..

[23] Ivanov G.R., Avramov I.D., Strijkova V.J., Marinov Y.G., Vlakhov T.E., Bogdanova E., Hadjichristov G.B. (2021). Mass sensitivity of Langmuir-Blodgett monolayer film coated surface acoustic wave resonators to volatile organic solvents. J. Phys. Conf. Ser..

[24] Ivanov G.R., Avramov I.D. (2019). Langmuir-Blodgett Films from Fluorescently Labelled Phospholipids Deposited on Surface Acoustic Wave Devices. J. Phys. Conf. Ser..

[25] Ivanov G.R., Polevska Z. (2017). First observation of 3D aggregates in a single-component Langmuir film below the equilibrium spreading pressure. MATEC Web Conf..

[26] Ivanov G.R., Kostadinov K.G., Song Z. Formation of nanosized needle structures in ultra-thin organic film for biosensor application. Proceedings of the IEEE International Conference on Manipulation, Manufacturing and Measurement on the Nanoscale.

[27] Chang Y., Tang N., Qu H., Liu J., Zhang D., Zhang H., Pang W., Duan X. (2016). Detection of volatile organic compounds by self-assembled monolayer coated sensor array with concentration-independent fingerprints. Sci. Rep..

[28] Brondz I. (2005). Fatty Acids. Reference Module in Chemistry, Molecular Sciences and Chemical Engineering.

[29] Deng Y., Xu L., Lu H., Wang H., Shi Y. (2018). Direct measurement of the contact angle of water droplet on quartz in a reservoir rock with atomic force microscopy. Chem. Eng. Sci..

[30] Erb R.A. (1968). Wettability of gold. J. Phys. Chem..

[31] Gretic Z.H., Mioc E.K., Cadez V., Segota S., Curkovic H.O., Hosseinpour S. (2016). The Influence of Thickness of Stearic Acid Self-Assembled Film on Its Protective Properties. J. Electrochem. Soc..

[32] Oncins G., Torrent-Burgues J., Sanz F. (2008). Nanomechanical Properties of Arachidic Acid Langmuir-Blodgett Films. J. Phys. Chem. C.

[33] Nečas D., Klapetek P., Valtr M. (2020). Estimation of roughness measurement bias originating from background subtraction. Meas. Sci. Technol..

[34] Nečas D., Valtr M.M., Klapetek. P. (2020). How levelling and scan line corrections ruin roughness measurement and how to prevent it. Sci. Rep..

[35] Avramov I.D. (2000). High-performance surface transverse wave resonators in the lower GHz frequency range. Int. J. High Speed Electron. Syst..

[36] Avramov I.D. Design of Rayleigh SAW resonators for applications as gas sensors in highly reactive chemical environments. Proceedings of the 2006 IEEE International Frequency Control Symposium and Exposition.

[37] Cross P.S., Shreve W.R. Synchronous IDT SAW resonators with Q above 10,000. Proceedings of the 1979 Ultrasonics Symposium.

[38] Avramov I.D., Voigt A., Rapp M. (2005). Rayleigh SAW resonators using gold electrode structure for gas sensor applications in chemically reactive environments. Electron. Lett..

[39] Martin S.J., Ricco A.J., Niemczyk T.M., Frye G.C. (1989). Characterization of SH acoustic plate mode liquid sensors. Sens. Actuators.

[40] Adamson A.W., Gast A.P. (1997). Physical Chemistry of Surfaces.

[41] Hansen C.M. (2007). Hansen Solubility Parameters: A User’s Handbook.

[42] Barton A. (1983). Handbook of Solubility Parameters and Other Cohesion Parameters.

[43] Lee W.J., Goh P.S., Lau W.J., Ismail A.F., Hilal H. (2021). Green Approaches for Sustainable Development of Liquid Separation Membrane. Membranes.

